# AC-PCoA: Adjustment for confounding factors using principal coordinate analysis

**DOI:** 10.1371/journal.pcbi.1010184

**Published:** 2022-07-13

**Authors:** Yu Wang, Fengzhu Sun, Wei Lin, Shuqin Zhang

**Affiliations:** 1 School of Mathematical Sciences, Fudan University, Shanghai, China; 2 Research Institute of Intelligent Complex Systems, Fudan University, Shanghai, China; 3 Quantitative and Computational Biology Department, University of Southern California, Los Angeles, California, United States of America; 4 State Key Laboratory of Medical Neurobiology, MOE Frontiers Center for Brain Science, and Institutes of Brain Science, Fudan University, Shanghai, China; 5 Shanghai Artificial Intelligence Laboratory, Shanghai, China; 6 Key Laboratory of Mathematics for Nonlinear Science (Fudan University), Ministry of Education, Shanghai, China; 7 Shanghai Key Laboratory for Contemporary Applied Mathematics (Fudan University), Shanghai, China; Univrsity of Pittsburgh, UNITED STATES

## Abstract

Confounding factors exist widely in various biological data owing to technical variations, population structures and experimental conditions. Such factors may mask the true signals and lead to spurious associations in the respective biological data, making it necessary to adjust confounding factors accordingly. However, existing confounder correction methods were mainly developed based on the original data or the pairwise Euclidean distance, either one of which is inadequate for analyzing different types of data, such as sequencing data.

In this work, we proposed a method called Adjustment for Confounding factors using Principal Coordinate Analysis, or AC-PCoA, which reduces data dimension and extracts the information from different distance measures using principal coordinate analysis, and adjusts confounding factors across multiple datasets by minimizing the associations between lower-dimensional representations and confounding variables. Application of the proposed method was further extended to classification and prediction. We demonstrated the efficacy of AC-PCoA on three simulated datasets and five real datasets. Compared to the existing methods, AC-PCoA shows better results in visualization, statistical testing, clustering, and classification.

## Author summary

With today’s unprecedented amount of data, researchers are challenged by the need to enhance meaningful signals without the interference of unwanted confounders hidden inside the data. Data visualization is an important step toward exploring and explaining data in order to intuitively identify the dominant patterns. Principal coordinate analysis (PCoA), as a visualization tool, allows flexible ways to define pairwise distances and project the samples into lower dimensions without changing the distances. However, when visualizing large-scale biological datasets, the true patterns are often hindered by unwanted confounding variations, either biologically or technically in origin. To eliminate these confounding factors and recover underlying signals, we proposed a method called Adjustment for Confounding factors using Principal Coordinate Analysis, or AC-PCoA, and showed that it significantly outperforms existing methods in visualization through three simulation studies and five real datasets. We further showed that the low-dimensional representations given by AC-PCoA provide promising results in statistical testing, clustering, and classification as well.

This is a *PLOS Computational Biology* Methods paper.

## Introduction

Confounding factors, which are generally regarded as hidden variables, exist widely in various biological data, and they affect data in unknown ways. Some of these confounding factors are caused by technical issues, also known as batch effects, such as lab variations in multisite data generation processes. Others are biologically oriented, such as unwanted differences of sex, age, or ethnic groups. Such factors may mask the true signals and lead to spurious findings. Therefore, it is necessary to correct the confounding factors when analyzing datasets with possible underlying confounders.

Many methods have been developed in the last few decades to remove confounding factors directly. For example, Johnson *et al*. [1] proposed parametric and non-parametric empirical Bayes methods, which are robust to outliers for samples of small size, to adjust for batch effects. Leek *et al*. [2–4] introduced surrogate variable analysis (SVA) for identifying, estimating, and incorporating sources of expression heterogeneity into gene expression analysis. SVA identifies groups of genes affected by each unobserved factor and estimates the factor based on the expression of those genes. Negative controls and technical replicates have also been introduced to identify and remove unwanted variations in high-dimensional data [5–8]. A large number of scientific research from various disciplines are still focusing on this issue in recent years [9–14].

More high-dimensional data lead to more corresponding demand for simultaneous dimension reduction and confounding factor correction. To meet this demand, Lin *et al*. [15] proposed AC-PCA for simultaneous dimension reduction and adjustment for confounding variations. It is a model-free method, and it has shown good performance when removing variations across individual donors in a human brain exon array dataset and across different species in an ENCODE RNA-Seq dataset. However, when only pairwise distances are available in the data, AC-PCA is no longer applicable. In reality, there are also situations where non-Euclidean distances are better at describing pairwise relationships. For example, alignment-free distances [16–18] are particularly designed for next-generation sequencing data, and Bray-Curtis distance [19] is widely used in the field of metagenomics, while Manhattan distance is suitable for data sampled from Laplace distribution. Some generalized distance measures are specifically designed for ordinal data [20], categorical data [21], and sparse data [22, 23]. Involving analysis of the proper distance measures can help capture major, as well as subtle, differences among samples. Such cases require appropriate methods to adjust for confounding variation.

Principal Coordinate Analysis (PCoA), also known as classical Multidimensional Scaling (MDS), is a popular method of dimension reduction when only the distance measures are given. It was seminally proposed by Torgerson in 1958 [24] and Gower in 1966 [25], and it has been widely used in biological and ecological studies [26–28]. Based on PCoA, adjusted Principal Coordinates Analysis (aPCoA) is a recently proposed method for adjusting covariates [29]. By calculating the eigenvectors and the eigenvalues of confounder-adjusted Gower’s center matrix, aPCoA can improve data visualization and enhance presentation of the effects of interest. However, aPCoA assumes a linear relationship between the Euclidean representation of data and the confounding factors, which may introduce bias and remove desired signals in the original data.

Therefore, in this work, we introduce AC-PCoA, a novel method to simultaneously perform dimension reduction and confounding factor removal. This method definitely can manage a large variety of confounders for various types of data and distances. AC-PCoA can also be extended for data preprocessing in classification and prediction problems when confounding factors exist. In order to further validate the performance of AC-PCoA, we consider four evaluation criteria, using three simulated datasets and five real datasets. Then, comparisons with the existing methods show that AC-PCoA gives more meaningful patterns in visualization, more significant results in MANOVA testing, as well as better clustering and classification accuracy.

## Methods

In this section, we first review AC-PCA, and then present AC-PCoA in detail. Furthermore, we discuss the applications of AC-PCoA in classification problems.

### AC-PCA

AC-PCA was proposed by Lin *et al*. [15] to perform simultaneous dimension reduction and adjustment for confounding variations. In a typical case, let *X* be an *N* × *p* data matrix representing *N* samples and *p* features with each data point denoted as ***x***_*i*_ ∈ *R*^*p*^. Here, *X* is centered by column. Let *Y* be the *N* × *l* matrix for *l* confounding factors with ***y***_*i*_ ∈ *R*^*l*^ as the confounding factor of each sample ***x***_*i*_. AC-PCA modifies principal component analysis (PCA) and aims to solve the following optimization problem:

$$\begin{array}{c} \begin{array}{cc} & \underset{V}{\text{max}}\;\;\text{trace}\{V^{⊤}X^{⊤}XV-\lambda V^{⊤}X^{⊤}KXV\},\\ & s.t.\;\;∥v_{t}{∥}_{2}\leq 1,\;v_{t}^{⊤}v_{g}=0,\;t,g=1,2,\ldots ,T,\;t\neq g, \end{array} \end{array}$$ (1)

where ***v***_*t*_ and ***v***_*g*_ denote the *t*-th and *g*-th columns of *V*, and *T* is the reduced dimensionality. In addition, *K* is the *N* × *N* kernel matrix constructed from the confounding factors, and *K*_*ij*_ = *k*(***y***_*i*_, ***y***_*j*_). The first term in the objective function maximizes the variance of the projected data *XV*, as in principal component analysis. The second term penalizes the dependence between projected data *XV* and the confounding factors *Y*. The parameter λ balances these two terms. Denote *Z* = *X*^⊤^*X* − λ*X*^⊤^*KX*. The above optimization problem can be rewritten as:

$$\begin{array}{c} \begin{array}{cc} & \underset{V}{\text{max}}\;\;\text{trace}\{V^{⊤}ZV\},\\ & s.t.\;\;∥v_{t}{∥}_{2}\leq 1,\;v_{t}^{⊤}v_{g}=0,\;t,g=1,2,\ldots ,T,\;t\neq g. \end{array} \end{array}$$

It is straightforward to solve this optimization problem by implementing an eigen-decomposition on *Z*.

AC-PCA is effective when Euclidean distance is used to characterize sample relationships. However, it is a common case in biological data that non-Euclidean distance is better for describing pairwise dissimilarities. Accordingly, we were motivated to extend AC-PCA to AC-PCoA for handling more generalized distance measures.

### AC-PCoA: Confounding factor adjustment based on pairwise distances

In this subsection, we extend PCoA to AC-PCoA to perform confounding factor adjustment with dimension reduction. As previously noted, PCoA is a popular dimension reduction and visualization method when pairwise distances of the samples are given without the original data. It projects the samples into a lower-dimensional Euclidean space so that the given pairwise relations are preserved. The procedure of applying PCoA can be summarized in the following steps:

1. Given the *N* × *p* data matrix *X*, representing *N* samples and *p* features, the pairwise distance matrix *D* using the desired distance measure is first calculated. If data available are pairwise distance matrix *D*, go to the next step.
2. Transform distance matrix to similarity matrix *A*: $a_{ij}=-\frac{1}{2}d_{ij}^{2}$.
3. Normalize similarity matrix: $\hat{A}=(I-1s^{⊤})A(I-s1^{⊤})$, where $s=\frac{1}{N}1$ and **1** = (1, …, 1)^⊤^.
4. Calculate the *M* eigenvectors corresponding to the *M* leading eigenvalues λ_*m*_, *m* = 1, 2, …, *M* of $\hat{A}$. These eigenvectors are then normalized to have norm $\sqrt{\lambda_{m}}$.

The result of PCoA is defined as matrix ${\hat{X}}_{M}$ with each column being one of the *M* corresponding normalized eigenvectors of $\hat{A}$. In the following, we set *M* as the number of positive eigenvalues of $\hat{A}$, which can well capture the patterns in the data, and simplify ${\hat{X}}_{M}$ as $\hat{X}$. Notice that PCoA is equivalent to PCA when Euclidean distance is used to calculate the pairwise distances [30]. Detailed explanations were given in Gower [25, 31] and Legendra [24].

To extend PCoA to handle multiple datasets with confounding factors, we aim to preserve pairwise distances in a lower dimensional space, and at the same time minimize the associations between the lower-dimensional representation and the confounding variables. Based on principal coordinate representations $\hat{X}$ of the original data, AC-PCoA is proposed as a method of adjusting confounding factors that finds the principal directions by solving the following optimization problem:

$$\begin{array}{c} \begin{array}{cc} & \underset{V}{\text{max}}\;\;\text{trace}\{V^{⊤}{\hat{X}}^{⊤}\hat{X}V-\lambda V^{⊤}{\hat{X}}^{⊤}K\hat{X}V\},\\ & s.t.\;\;∥v_{t}{∥}_{2}\leq 1,\;v_{t}^{⊤}v_{g}=0,\;t,g=1,2,\ldots ,T,\;t\neq g, \end{array} \end{array}$$ (2)

where the notations are the same as those in AC-PCA. Confounding factors are user-defined and depend on the assumptions of confounding factors’ variation. We provide several examples on the choice of *Y* in simulation studies and real data analysis. To solve the optimization problem (2), we denote $\hat{Z}={\hat{X}}^{⊤}\hat{X}-\lambda {\hat{X}}^{⊤}K\hat{X}$, and the optimization problem can be rewritten as:

$$\begin{array}{c} \begin{array}{cc} & \underset{V}{\text{max}}\;\;\text{trace}\left(V^{⊤}\hat{Z}V\right),\\ & s.t.\;\;∥v_{t}{∥}_{2}\leq 1,\;v_{t}^{⊤}v_{g}=0,\;t,g=1,2,\ldots ,T,\;t\neq g. \end{array} \end{array}$$

By implementing eigen-decomposition on $\hat{Z}$, we may get the principal directions $\hat{V}$ and the data representation $\hat{X}\hat{V}$. Note that PCoA is equivalent to PCA when Euclidean distance is used to calculate pairwise distances. We performed extensive simulation studies and real data analysis, and the experiments showed that the results of AC-PCoA, when using Euclidean distance, are pretty close to that of AC-PCA.

As for the choice of parameter λ, we followed Lin *et al*. [15] and defined $R\left(\lambda \right)=\frac{\text{trace}(V^{⊤}{\hat{X}}^{⊤}K\hat{X}V)}{\text{trace}(V^{⊤}{\hat{X}}^{⊤}\hat{X}V)}$ to be the ratio of penalty term verses the principal projection. As λ increases from 0, *R*(λ) tends to decrease. When the penalty term in Eq (2) is designed as the between-groups sum of squares, λ is determined by the smallest value such that *R*(λ) ≤ 0.05 in the principal coordinates of interest. For other definitions of the penalty term, we choose the smallest λ that can satisfy *R*(λ) ≤ 0.05*R*(0). It is worth mentioning that the overall results are quite robust against the fluctuation of λ in a wide range.

### Data preprocessing using AC-PCoA in classification and prediction problems

In this subsection, we extend the application of AC-PCoA to classification and prediction. In large-scale data analysis, the data may be collected from multiple sites or different groups, which could affect the performance of prediction methods. Correcting these confounding factors can help improve prediction accuracy. Here, we adapt AC-PCoA to correct confounding factors and perform dimension reduction for the training data and test data, and then conduct prediction and classification.

Suppose we have training dataset {*X*, *Y*, **z**}, where *X* is the covariate data matrix of size *N* × *p* with each data point denoted as ***x***_*i*_ ∈ *R*^*p*^, *Y* is the *N* × *l* confounding factor matrix with ***y***_*i*_ ∈ *R*^*l*^ as the confounding factors of each sample ***x***_*i*_, and **z** describes the classes to which each sample belongs for *i* = 1, 2, ⋯, *N*. The relationships between *X* and **z** are modelled such that the corresponding class of a new data point ***x*** can be predicted. When confounding factors *Y* exist, the prediction may be misled by these variations. However, by applying AC-PCoA to the training set, we can obtain the lower-dimensional representations $\hat{X}\hat{V}\text{s}$ after confounder adjustment and then we can train the classification and prediction model. When a test data point ***x*** ∈ *R*^*p*^ with confounding factor ***y*** ∈ *R*^*l*^ comes, higher prediction accuracy is expected by using the data point’s lower-dimensional representation $\hat{x}\hat{V}$ in the same space as that of the training data for classification.

We employ the idea of kernel PCA [32] to perform confounder correction for the newcoming data point ***x***. Consider a feature space introduced by a mapping Φ(⋅), which is implicit and is characterized by a kernel matrix $\hat{A}\left(x_{i},x_{j}\right)=\langle \Phi \left(x_{i}\right),\Phi \left(x_{j}\right)\rangle$, where $\hat{A}$ is the normalized similarity matrix in PCoA. For the training data, PCoA is equivalent to projecting the mapped data Φ(***x***_*i*_) onto the direction of the first *M* normalized eigenvectors ***w***_1_, ⋯, ***w***_*M*_ of the covariance matrix $C=\frac{1}{N}\sum_{j=1}^{N}\Phi \left(x_{j}\right)\Phi {\left(x_{j}\right)}^{⊤}$, i.e., for each ${\hat{x}}_{i}$, ${\left\{{\hat{x}}_{i}\right\}}_{m}=\langle w_{m},\Phi \left(x_{i}\right)\rangle$, where ‖***w***_*m*_‖_2_ = 1, *m* = 1, …, *M*. This projection becomes tractable since $\langle w_{m},\Phi \left(x_{i}\right)\rangle =\langle u_{m},\hat{A}\left(x_{i},\cdot \right)\rangle$, where ***u***_*m*_ = (*u*_*m*1_, ⋯, *u*_*mn*_) is the eigenvector of kernel matrix $\hat{A}$ with norm $\frac{1}{\sqrt{\lambda_{m}}}$ corresponding to the *m*-th eigenvalue λ_*m*_ of $\hat{A}$. For a test point ***x***, the image of which is Φ(***x***), one can also use the same idea and compute the projected point $\hat{x}$, where ${\left\{\hat{x}\right\}}_{m}=\langle w_{m},\Phi \left(x\right)\rangle =\langle u_{m},\hat{A}\left(x,\cdot \right)\rangle$. $\hat{A}\left(x,\cdot \right)$ denotes the vector of centered kernel function applied to ***x*** and all training points. After obtaining the projected test data $\hat{x}$ from PCoA, we then adjust for confounding factors by multiplying the projection $\hat{x}$ with the direction $\hat{V}$ obtained from the training step. We can then perform classification on the lower-dimensional representations ${\hat{x}}^{⊤}\hat{V}$ of the test data points. The procedure is given below.

1. Perform AC-PCoA on training data *X* to get principal direction matrix ${\hat{V}}_{T}=\left({\hat{v}}_{1},\cdots ,{\hat{v}}_{T}\right)$ and data projection matrix ${\hat{X}}_{T}=\hat{X}{\hat{V}}_{T}$. Meanwhile, save matrix $\hat{A}$ as similarity matrix and matrix *U* = (***u***_1_, ***u***_2_, …, ***u***_*M*_) as the matrix of eigenvectors of $\hat{A}$ corresponding to positive eigenvalues with $∥u_{m}{∥}_{2}=\frac{1}{\sqrt{\lambda_{m}}}$ for later use.
2. Conduct AC-PCoA on test data.
  - For a test data point ***x***, calculate the distances between it and all the training data points. Denote this vector as *D*(***x***, ⋅) = (*d*_***x***1_, ⋯, *d*_***x**N*_).
  - Calculate the corresponding similarity vector *A*(***x***, ⋅) = (*a*_***x***1_, ⋯, *a*_***x**N*_) by $a_{xi}=-\frac{1}{2}d_{xi}^{2},\;i=1,\cdots N$. Center it to the same origin as that of the training data by $\hat{A}\left(x,\cdot \right)=A\left(x,\cdot \right)-\frac{1}{n}11^{⊤}A\left(x,\cdot \right)-\frac{1}{n}A1+\frac{1}{n}11^{⊤}A1$.
  - Calculate PCoA representation of test data point by $\hat{x}=U^{⊤}\hat{A}\left(x,\cdot \right)$.
  - Obtain the *T* dimensional AC-PCoA representation ${\hat{x}}_{T}$ by ${\hat{x}}_{T}={\hat{x}}^{⊤}{\hat{V}}_{T}$.
3. Use ${\hat{X}}_{T}$ as input to train the classifier.
4. Feed ${\hat{x}}_{T}$ to the trained classifier to do prediction.

By first projecting the data to a lower-dimensional space and selecting relevant features, signal-to-noise ratio can be increased, which might help improve classification accuracy. AC-PCoA, as a preprocessing step, is beneficial to finding desired principal directions of data without the potential misdirection of confounding variation, thus improving classification accuracy.

### Evaluation criteria

The performance of AC-PCoA is evaluated through four different criteria.

1. Visualization. By projecting the data to a two-dimensional space after using AC-PCoA and coloring the data with inherent features, check whether AC-PCoA can remove confounding variations and recover the underlying patterns hidden in the data.
2. Multivariate analysis of variance (MANOVA). MANOVA can evaluate the significance of groups defined by data representation after confounding factor adjustment and the underlying true labels. In MANOVA, an *F*-statistic is defined to access the mean rank of distance between samples in two groups, and a permutation test is employed to calculate the p-value. In this paper, the function ‘anosim’ in R package vegan is employed to calculate the *F*-statistic. As the *F*-statistic increases, the significance of the cluster increases.
3. Normalized mutual information (NMI) [33]. NMI is one of the popular evaluation metrics estimating clustering quality. After conducting *k*-means clustering on low-dimensional data representations given by AC-PCoA, NMI is employed to measure how well the low-dimensional representations of samples are clustered. NMI is calculated using the ‘NMI’ function in R package aricode. The number of clusters *k* in *k*-means is set to be the number of true labels.
4. Classification accuracy. AC-PCoA can be applied as the preprocessing step in classification problems. Random forest is used as the classifier and parameters are tuned using grid search. Five-fold cross validation is used to evaluate performance. All the classification procedures are performed by tuning parameters on the training set only and evaluating accuracy on independent validation set. In the following analysis, the numbers of principal components are set to 2 and 3 to demonstrate the performance of AC-PCoA as a visualization tool. The classification performance of AC-PCoA on higher dimensions is provided in S1 Fig and S1 Appendix.

## Results

In this section, AC-PCoA was first tested on three simulation studies and then on five real datasets.

In all experiments, AC-PCoA was carried out following Eq (2), and the linear kernel was chosen. Different distance measures were considered, the definitions of which are provided in S2 Appendix. For comparison, we also conducted PCoA and aPCoA [29] using the same distance measures. We demonstrated the results of AC-PCA implicitly via AC-PCoA using Euclidean distance, denoted as AC-PCoA(eu), in real data analysis because the two- and three-dimensional representations given by AC-PCA are equivalent to those given by AC-PCoA(eu). Also, running AC-PCA takes more time than running AC-PCoA when the number of variables is large. Four criteria were employed to evaluate the performance of PCoA, AC-PCoA, and aPCoA. Note that aPCoA cannot be applied to classification.

### Simulation studies

We evaluated AC-PCoA in three simulation settings.

#### Setting 1

We simulated biological samples of different types generated from independent labs. For samples from lab *i*, we assumed that the data matrix was generated from *X*_*i*_ = *F*(Ω + *α*Γ^(*i*)^ + *ϵ*^(*i*)^), where Ω is the low rank component shared among labs, Γ^(*i*)^ is the lab-specific component, *α* represents the strength of confounding variation, and *ϵ*^(*i*)^ is Gaussian noise. The lab-specific variation is modeled as $\Gamma^{\left(i\right)}=\Lambda_{1}^{\left(i\right)}+\Lambda_{2}^{\left(i\right)}$. In $\Lambda_{1}^{\left(i\right)}$, the lab’s effect is the same in all samples within one lab. In $\Lambda_{2}^{\left(i\right)}$, the lab’s effect is different in that only a subset of samples is affected, allowing for more complicated confounding effects. By stacking the rows of *X*_*i*_, we formed a matrix *X* representing the data from all labs.

Specifically, samples of 3 different types were generated from 5 independent labs. Each lab contains *n* = 9 samples, among which 3 samples belong to the same type. The length of variables in each sample is *p* = 400. *F*(⋅) is a nonlinear element-wise function with F(·)=exp(·). For visualization, we assumed that the shared component Ω = *EH* has rank 2. *E* = (*e*_1_, *e*_2_) is an *n* × 2 matrix, representing the latent structure of the shared variation. We further assumed that samples of the same type have the same low rank representation. That is, 3 distinct rows comprise *E*, corresponding to samples of 3 different types. Each entry is generated from Uniform[−3, 3]. The rows of *E* corresponding to samples of the same type have the same values, *H* is a 2 × *p* matrix, and the rows in *H* are generated from $N(0,I_{p})$. For the lab-specific component $\Gamma^{\left(i\right)}=\Lambda_{1}^{\left(i\right)}+\Lambda_{2}^{\left(i\right)}$, we set $\Lambda_{1}^{\left(i\right)}=1r_{i}$, and $\Lambda_{2}^{\left(i\right)}=B_{i}s_{i}$. Here, *B*_*i*_ is an *n* × 1 matrix, wherein three random entries are generated from Uniform[0, 2], and the other entries are set to 0. Moreover, *r*_*i*_ and *s*_*i*_ are 1 × *p* matrices generated from $N(0,I_{p})$, and *α* is set to be 2.5. Each row of *ϵ*^(*i*)^ is generated from $N(0,0.25I_{p})$.

To pool samples from multiple labs together, we performed AC-PCoA using Eq (2) to remove lab-specific component Γ^(*i*)^ and capture the shared component Ω. Confounding factor matrix *Y* in Eq (2) is defined to be a matrix of *N* × 10, wherein each column has two groups of non-zero entries, $\frac{1}{n}$ corresponding to the samples from lab *i* and $-\frac{1}{n}$ corresponding to those from lab *j*. Hence, the optimization problem (2) becomes:

$$\begin{array}{ccc} & & \underset{V}{\text{max}}\;\;\text{trace}\{V^{⊤}{\hat{X}}^{⊤}\hat{X}V-\lambda \sum_{i=1}^{4}\sum_{j=i+1}^{5}V^{⊤}{\left[f\left({\hat{X}}_{j}\right)-f\left({\hat{X}}_{i}\right)\right]}^{⊤}[f\left({\hat{X}}_{j}\right)-f\left({\hat{X}}_{i}\right)]V\},\\ & & s.t.\;\;∥v_{t}{∥}_{2}\leq 1,\;v_{t}^{⊤}v_{g}=0,\;t,g=1,2,\ldots ,T,\;t\neq g, \end{array}$$

where $f\left({\hat{X}}_{i}\right)=\frac{1}{n}1^{⊤}{\hat{X}}_{i}$.

**Fig 1:**
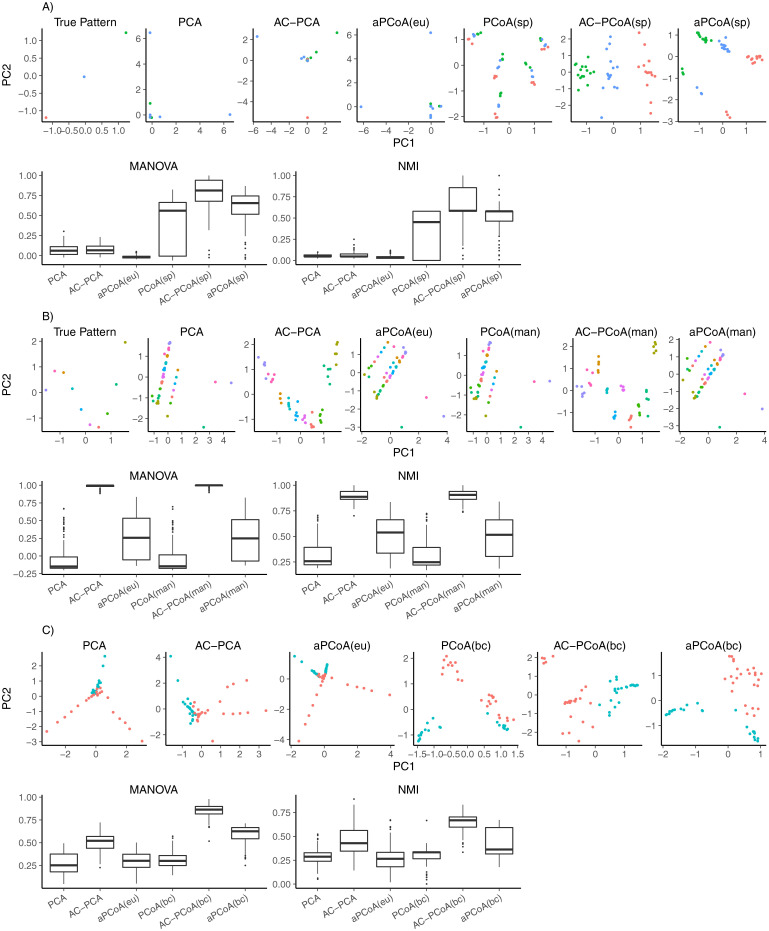
Results of simulation data. A: Simulation setting 1. The first line shows the true pattern and two-dimensional representations of samples from PCA, AC-PCA, PCoA(sp), AC-PCoA(sp) and aPCoA for one representative run. Samples are colored according to 3 types. The second line shows box plots of MANOVA *F*-statistic and NMI of *k*-means clustering on two-dimensional representations for 100 runs. B: Simulation setting 2. The first line shows the true pattern and two-dimensional sample representations from PCA, AC-PCA, PCoA(man), AC-PCoA(man) and aPCoA for one representative run. Samples are colored according to 10 types. The second line shows box plots of MANOVA *F*-statistic and NMI of *k*-means clustering for 100 runs. C: Simulation setting 3. The first line shows two-dimensional sample representations from PCA, AC-PCA, PCoA(bc), AC-PCoA(bc) and aPCoA for one representative run. Samples are colored according to 2 clinical groups. The second line shows box plots of MANOVA *F*-statistic and NMI of *k*-means clustering for 100 runs.

The results of one representative run and 100 runs are shown in Fig 1A. Note that the nonlinear function in this setting is a monotonically increasing function. We selected Spearman distance (sp) as the distance measure in AC-PCoA because Spearman distance only takes the order of data into consideration. The results of visualization, MANOVA, and NMI all show that AC-PCoA(sp) has sufficient flexibility to manage nonlinear structures.

#### Setting 2

Under the same framework as that for setting 1, parameters of the second simulation setting are given below. Samples of 10 different types were generated from 5 independent labs. Each lab contains *n* = 10 samples of different types. The length of variables of each sample is *p* = 400. *E* = (*e*_1_, *e*_2_) is an *n* × 2 matrix. Define *μ* = (1, ⋯, *n*)^⊤^ and scale *μ* to have mean 0 and variance 1. Particularly, *e*_1_ is set to be the scaled *μ*, and *e*_2_ is assumed to be sampled from multivariate Laplace distribution Laplace(**0**, 0.25*Σ*), where Σ_*ij*_ = exp [−(*e*_*i*1_ − *e*_*j*1_)^2^/4]. Additionally, *H* is a 2 × *p* matrix and its rows are generated from multivariate Laplace distribution Laplace(**0**, *I*_*p*_). The lab-specific components are set to be $\Lambda_{1}^{\left(i\right)}=1r_{i}$ and $\Lambda_{2}^{\left(i\right)}=B_{i}s_{i}$. Here, each entry of *B*_*i*_ is generated from Uniform[0, 2], *r*_*i*_ and *s*_*i*_ are generated from multivariate Laplace distribution Laplace(**0**, *I*_*p*_), *α* is set to be 2.5, and the entries in *ϵ*^(*i*)^ are generated from Laplace(0, 0.25) independently.

Results of setting 2 are shown in Fig 1B. Because Manhattan distance (man) is commonly used to describe pairwise distances of samples generated from Laplace distribution, we implemented AC-PCoA using Manhattan distance. We set *Y* to have the following structure: each column of *Y* contains only two non-zero entries, 1 and −1, corresponding to the rows of a pair of samples of the same type, but from different labs. Thus, Eq (2) becomes:

$$\begin{array}{ccc} & & \underset{V}{\text{max}}\;\;\text{trace}\{V^{⊤}{\hat{X}}^{⊤}\hat{X}V-\frac{\lambda }{5}\sum_{i=1}^{4}\sum_{j=i+1}^{5}V^{⊤}{\left({\hat{X}}_{j}-{\hat{X}}_{i}\right)}^{⊤}\left({\hat{X}}_{j}-{\hat{X}}_{i}\right)V\},\\ & & s.t.\;\;∥v_{t}{∥}_{2}\leq 1,\;v_{t}^{⊤}v_{g}=0,\;t,g=1,2,\ldots ,T,\;t\neq g. \end{array}$$

The results show that AC-PCoA(man) gives better visualization results compared to AC-PCA since samples of the same label are clustered more tightly. AC-PCoA(man) also outperforms AC-PCA in both MANOVA and NMI.

#### Setting 3

We used the ‘SimulateMSeq’ function in R package GUniFrac [34] to simulate microbiome data from Dirichlet-multinomial distribution. GUniFrac is a popular R-package for microbiome data. ‘SimulateMSeq’ implements a semiparametric approach and generates synthetic microbiome sequencing data to study the performance of different abundance analysis methods. We simulated samples from 2 clinical groups, each of which contains *n* = 25 samples. The number of OTUs was set to be *p* = 100. 80% of the OTUs were affected by the label of clinical groups. We further assumed that samples were collected by 2 independent labs where 80% of OTUs were affected by batch labeling.

The results are shown in Fig 1C. Since Bray-Curtis distance (bc) is commonly applied to microbiome abundance data, we implemented AC-PCoA with Bray-Curtis distance. Confounding factors are chosen in the same manner as that in Setting 1. Fig 1C shows that the performance of AC-PCoA(bc) is better than that of the other methods.

### Real data analysis

In this section, we applied the proposed method to five real datasets to evaluate its performance: 1) whole genome shotgun sequencing data of white oak trees, 2) human microbiome OTU counts table from the Microbiome Quality Control Project, 3) RNA-Seq data from the Sequencing Quality Control Project, 4) single-cell RNA-Seq data of human PBMCs, and 5) human brain exon array data.

#### NGS whole genome shotgun sequencing data of white oak trees

We first applied AC-PCoA to NGS whole genome shotgun (WGS) sequencing data of white oak trees. Data were downloaded from NCBI BioProject PRJNA269970, PRJNA308314, and PRJNA327502. The samples in the first two BioProjects were collected using the Illumina platform. In the third BioProject, 8 samples were collected using Illumina, and 22 using PacBio. Owing to the small size and the outlier performance, nine data points were deleted [35]. After preprocessing, we were left with a total of 131 samples from 4 batches. Samples were divided into three geographic categories according to their continental origins. Samples from the United States and Canada were categorized as North America (NA). Samples from west of 100°*E* longitude were categorized as West Europe (WE). And samples from east of 100°*E* longitude were categorized as East Europe and Asia (EEA). The origins were considered as underlying true labels of the data. To reduce the effects caused by different sequence quantities, we downsampled the data to produce random samples of reads totaling 100 Mbp for each sample. We took the unwanted variations between different BioProjects and sequencing platforms as confounding factors.

Note that the original data are raw sequence reads, to which most computational methods, including PCA and AC-PCA, cannot be applied. Here, we employed six alignment-free distance measures specifically designed for next generation sequencing data, including three traditional distance measures: Manhattan distance (man), Euclidean distance (eu), and *d*_2_ distances (d2), as well as three recently developed background-adjusted measures: CVTree, $d_{2}^{*}$ (d2star) and $d_{2}^{s}$ (d2shepp). These distances are based on the relative frequencies of k-mers (k-grams, k-tuples, k-words). Here, k-mer length is set to be 12 and Markov order is set to be 10.

Denote by *E*_*i*_ the set of tree sequences from batch *i*, where *i* = 1, …, 4. Suppose *n*_*i*_ be the number of trees from batch *i*. Let *E* represent the whole set of tree sequences from all batches. Further assume *N* = *n*_1_ + ⋯ + *n*_4_ as the total number of trees. Confounding factor matrix *Y* in Eq (2) is defined to be a matrix of *N* × 6, wherein each column has two groups of non-zero entries, $\frac{1}{n_{i}}$ corresponding to the samples from batch *i* and $-\frac{1}{n_{j}}$ corresponding to those from batch *j*. Hence, the optimization problem (2) becomes:

$$\begin{array}{ccc} & & \underset{V}{\text{max}}\;\;\text{trace}\{V^{⊤}{\hat{X}}^{⊤}\hat{X}V-\lambda \sum_{i=1}^{3}\sum_{j=i+1}^{4}V^{⊤}{\left[f\left({\hat{X}}_{j}\right)-f\left({\hat{X}}_{i}\right)\right]}^{⊤}[f\left({\hat{X}}_{j}\right)-f\left({\hat{X}}_{i}\right)]V\},\\ & & s.t.\;\;∥v_{t}{∥}_{2}\leq 1,\;v_{t}^{⊤}v_{g}=0,\;t,g=1,2,\ldots ,T,\;t\neq g, \end{array}$$

where $f\left({\hat{X}}_{i}\right)=\frac{1}{n_{i}}1^{⊤}{\hat{X}}_{i}$.

**Fig 2:**
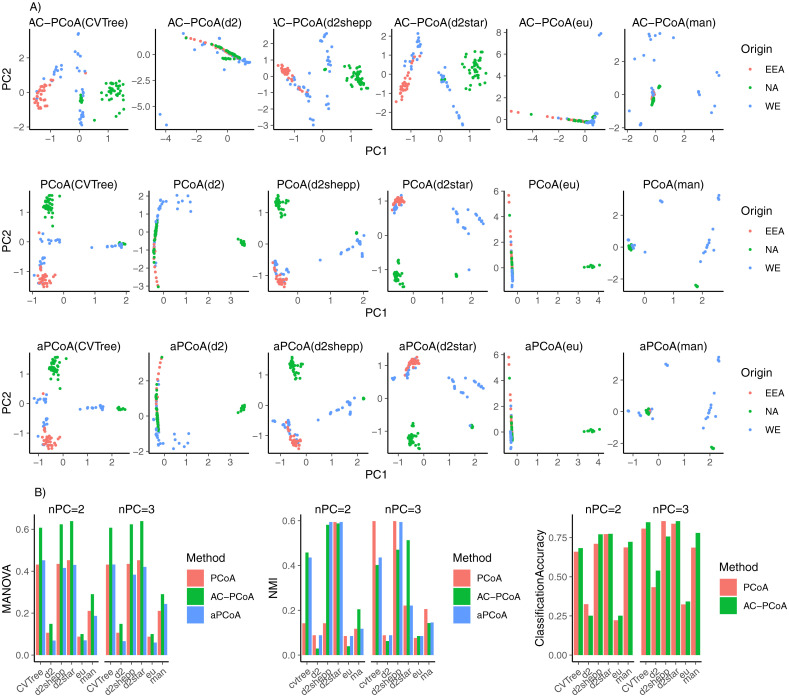
Results of white oak tree data. A: Two-dimensional representations of samples colored by continental origins after conducting AC-PCoA, PCoA, and aPCoA using six distance measures. B: MANOVA *F*-statistic, NMI of *k*-means clustering, and classification accuracy. Continental origins are set to be the true labels. MANOVA test, *k*-means clustering, and classification were conducted on two and three principal coordinates from PCoA, AC-PCoA, and aPCoA.

The results are shown in Fig 2. AC-PCoA demonstrates its superior ability to discriminate continental origins compared to that of either PCoA or aPCoA. Besides, $d_{2}^{*}$, $d_{2}^{s}$ and CVTree perform much better than traditional Euclidean distance. In MANOVA tests on both two and three dimensions, AC-PCoA outperforms PCoA and aPCoA under all six distance measures. NMI shows that three recently developed measures can better cluster trees from the same continental origin than traditional distances. AC-PCoA improves classification accuracy over that of PCoA under five out of six distance measures in both two and three dimensions by removing confounding factors in the data. These consistent results show that AC-PCoA can both remove confounding factors and contribute to downstream analysis.

#### The Microbiome Quality Control Project data

The Microbiome Quality Control Project (MBQC) [36] is a collaborative effort to comprehensively evaluate methods for measuring the human microbiome. Specifically, a set of initial samples of 23 specimens was collected. A subset of specimens was replicated or triplicated into 96-sample aliquot sets that were sent to 15 biology labs to carry out extraction and/or 16S amplicon sequencing. Each biology lab received one or more blinded copies of the 96-aliquot set. The raw sequence data were re-blinded and distributed to 9 bioinformatics labs for generating OTU counts of each sample. A total of 16140 samples were distributed in the final summarized data. We discarded samples without specimen information and samples with zero levels in all OTUs. Labs that processed fewer than 1000 samples were removed. Negative control samples were also removed. Thus, 16089 samples from 13 biology labs and 8 bioinformatics labs, including 22 specimens, were involved in the following analysis. Data were further grouped into 14 subsets (denoted as ‘A’, ‘B’, ‘C’, ‘D’, ‘E’, ‘F’, ‘1’, ‘2’, ‘3’, ‘4’, ‘5’, ‘6’, ‘7’, and ‘8’). Samples in subset ‘A’, ⋯, ‘F’ were processed by their own biology lab and different bioinformatics labs. Samples in subset ‘1’, ⋯, ‘8’ were processed by their own bioinformatics lab and different biology labs. The details of subset construction are described in S3 Appendix. This gave rise to 14 subsets in total. The following analyses were conducted on 14 subsets, respectively. The unwanted variations among different labs act as confounding factors in following analysis.

In the microbiome community, Bray-Curtis distance [19] is widely used to measure dissimilarity between samples, owing to the nature of abundance levels. In the following analysis, Bray-Curtis distance (bc) and Euclidean distance (eu) were implemented.

We employed subset ‘A’ as a demonstration. Let $X_{i}^{A}$ represent the *n* × *p* matrix for OTU levels of *n* samples and *p* OTUs processed by biology lab A and bioinformatics lab *i*. By stacking the rows of $X_{1}^{A},\ldots ,X_{8}^{A}$, we formed an *N* × *p* matrix *X*^A^ wherein *N* = 8 × *n*, representing the data from subset ‘A’. *Y* in Eq (2) was defined to have only two non-zero entries in each column, 1 and −1, corresponding to the rows of a pair of samples from the same specimen, but different labs. The optimization problem (2) was then formulated as:

$$\begin{array}{ccc} & & \underset{V}{\text{max}}\;\;\text{trace}\{V^{⊤}{\left({\hat{X}}^{A}\right)}^{⊤}{\hat{X}}^{A}V-\frac{\lambda }{8}\sum_{i=1}^{7}\sum_{j=i+1}^{8}V^{⊤}{\left[{\hat{X}}_{j}^{A}-{\hat{X}}_{i}^{A}\right]}^{⊤}[{\hat{X}}_{j}^{A}-{\hat{X}}_{i}^{A}]V\},\\ & & s.t.\;\;∥v_{t}{∥}_{2}\leq 1,\;v_{t}^{⊤}v_{g}=0,\;t,g=1,2,\ldots ,T,\;t\neq g. \end{array}$$

**Fig 3:**
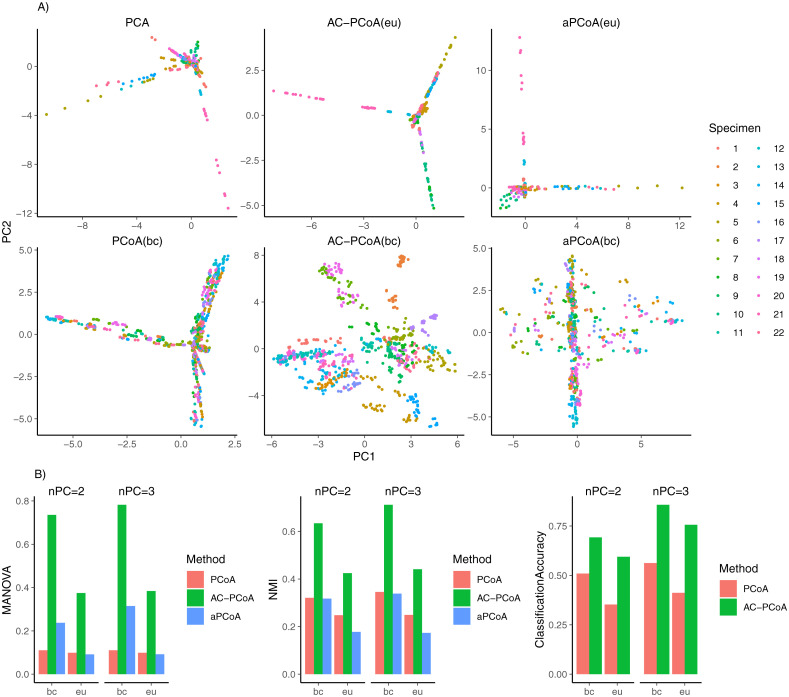
Results of MBQC data (Dataset ‘A’). A: Two-dimensional representations colored by specimens after conducting PCoA, AC-PCoA and aPCoA using Euclidean distance and Bray-Curtis distance. B: MANOVA *F*-statistic, NMI of *k*-means clustering, and classification accuracy. Specimens are set to be the true labels. MANOVA, *k*-means clustering, and classification were conducted on two and three principal coordinates from PCoA, AC-PCoA, and aPCoA.

Fig 3A shows the visualization results of subset ‘A’. Here, AC-PCoA(bc) distinguishes the original specimens better than all other methods in two-dimensional plots. AC-PCoA(eu) fails to give meaningful results because Euclidean distance is unable to describe dissimilarities between microbiome abundance levels. This example demonstrates the flexibility of AC-PCoA in handling non-Euclidean distance measures in order to facilitate visualization.

MANOVA, NMI and classification accuracy of subset ‘A’ are shown in Fig 3B. Results of all 14 subsets are shown in S2, S3 and S4 Figs. It is shown that AC-PCoA(bc) gives the highest MANOVA *F*-statistic and highest NMI in 13 out of 14 subsets in both two and three dimensions. Also, AC-PCoA(bc) gives the highest classification accuracy in 12 out of 14 subsets in both dimensions. This shows that AC-PCoA(bc) can cluster samples of the same specimen better and improve classification accuracy on two- and three-dimensional representations.

Moreover, we compared AC-PCoA with another popular data normalization method, SVA [3]. We conducted PCA after SVA for comparison. The results are included in S5 Fig. Results of SVA are not as good as those of AC-PCoA(bc) since it doesn’t take the proper pairwise relationships into account.

#### The Sequencing Quality Control Project data

The Sequencing Quality Control (SEQC) Project [37], also known as the third phase of the MAQC project (MAQC-III), is an FDA-led community-wide consortium aimed at assessing the technical performance of next-generation sequencing platforms at multiple sites by generating benchmark datasets with reference samples and evaluating advantages and limitations of various bioinformatics strategies in RNA and DNA analyses. Specifically, 6 distinguished reference samples (sample ID: A, B, C, D, E and F) were replicated and distributed to several independent sites for RNA-Seq library construction and profiling using three RNA-Seq platforms (Illumina HiSeq, Life Technologies SOLiD, and Roche 454). In this paper, we only consider data generated by six independent sites (NVS, COH, AGR, BGI, MAY and CNL) using Illumina HiSeq 2000. For simplicity, we only used data with the same replication number (i.e. replication number 1) in the following analysis. The variations caused by technical differences of six sites act as confounding factors.

In this dataset, we considered four distance measures: Euclidean distance (eu), Bray-Curtis distance (bc), Manhattan distance (man), and Spearman distance (sp). Let *X*_*i*_ represent the *n*_*i*_ × *p* matrix for the gene expression levels of *n*_*i*_ samples and *p* genes processed by site *i*. The sample size *n*_*i*_ is different for different sites owing to the different number of lanes and sectors conducted by independent sites. By stacking the rows of *X*_1_, ⋯, *X*_6_, we formed an *N* × *p* matrix *X* where *N* = *n*_1_ + ⋯+ *n*_6_. We defined *Y* to have only two groups of non-zero entries in each column, 1 and −1, corresponding to the rows of a pair of samples of the same reference sample IDs but from different sites. The optimization problem (2) was defined as:

$$\begin{array}{ccc} & & \underset{V}{\text{max}}\;\;\text{trace}\{V^{⊤}{\hat{X}}^{⊤}\hat{X}V-\frac{\lambda }{6}\sum_{i=1}^{5}\sum_{j=i+1}^{6}\sum_{l=1}^{6}V^{⊤}{\left[f\left({\hat{X}}_{jl}\right)-f\left({\hat{X}}_{il}\right)\right]}^{⊤}[f\left({\hat{X}}_{jl}\right)-f\left({\hat{X}}_{il}\right)]V\},\\ & & s.t.\;\;∥v_{t}{∥}_{2}\leq 1,\;v_{t}^{⊤}v_{g}=0,\;t,g=1,2,\ldots ,T,\;t\neq g, \end{array}$$

where *X*_*il*_ is a submatrix of *X*_*i*_, containing samples of reference sample ID *l* processed by site *i*, and $f\left({\hat{X}}_{il}\right)=1^{⊤}{\hat{X}}_{il}$.

**Fig 4:**
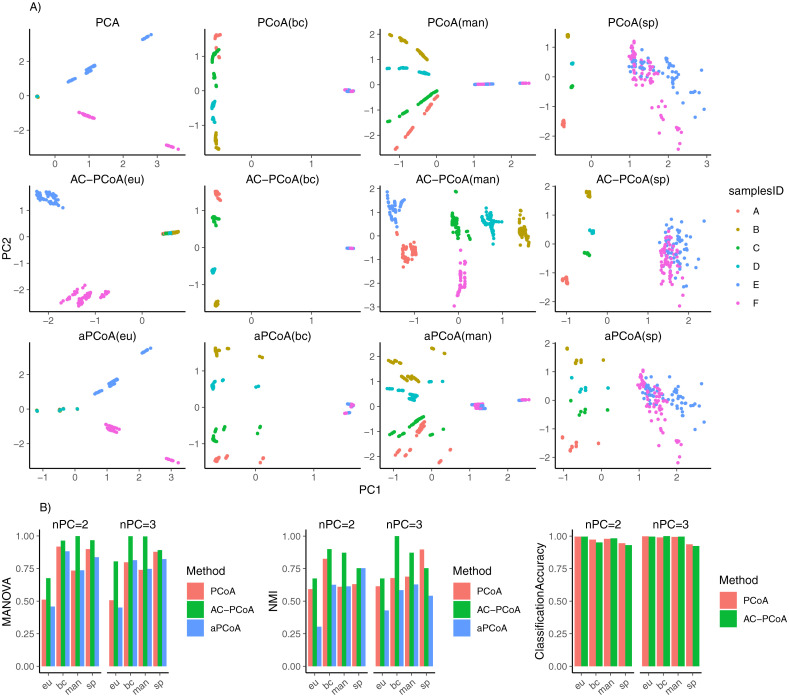
Results of SEQC data. A: Two-dimensional plots colored by reference sample IDs after conducting PCoA, AC-PCoA and aPCoA, using four distance measures. B: MANOVA *F*-statistic, NMI of *k*-means clustering, and classification accuracy. Reference samples IDs are set to be the true label. MANOVA test, *k*-means clustering, and classification were conducted on two and three principal coordinates from PCoA, AC-PCoA, and aPCoA.

Fig 4A shows that AC-PCoA can tightly cluster samples with the same reference sample ID compared to PCoA when the same distance measure is considered. aPCoA cannot improve clustering over PCoA. Euclidean distance is able to distinguish reference sample ID E and F, while the other three distances can separate reference sample ID A, B, C and D.

In Fig 4B, AC-PCoA gives higher MANOVA *F*-statistic than PCoA and aPCoA in all four distance measures in both two and three dimensions. AC-PCoA(bc) and AC-PCoA(man) show the best performance in clustering. These results demonstrate that incorporating non-Euclidean distances in confounding factor adjustment via AC-PCoA is necessary.

#### Single-cell RNA-Seq data

Single-cell experiments are often conducted with notable differences in capturing time, equipment and even technology platforms, which may introduce batch effects to the data. Up to now, it has remained challenging to characterize cell types across a wide variety of biological and technical conditions. We followed Korsunsky *et al*. [38] and gathered three datasets of human peripheral blood mononuclear cells (PBMCs), each of which assayed on the Chromium 10X platform but prepared with different protocols: 3’-end v1 (3pV1), 3’-end v2 (3pV2) and 5’-end (5p) chemistries. After pooling all the cells together, 6 cell types were identified in total. Since the number of cells of type “mk” was much smaller than that of the other 5 cell types, we discarded cell type “mk” and saved the other cell types (“bcells”, “dc”, “mono”, “nk” and “tcells”) for later analysis. To simplify computation, we then randomly selected at most sixty cells from each cell type and each protocol, and constructed a subset consisting of 849 cells. Afterwards, we normalized the data following [38] and performed the analysis on the normalized expression matrix.

We considered Euclidean distance (eu), Bray-Curtis distance (bc), Manhattan distance (man), and Spearman distance (sp). Let *X*_*i*_ represent the *n*_*i*_ × *p* matrix for the normalized expression level of *n*_*i*_ cells and *p* genes processed by the *i*-th protocol. We stacked the rows of *X*_1_, *X*_2_, *X*_3_, and formed an *N* × *p* matrix *X* of the pooled data wherein *N* = *n*_1_ + *n*_2_ + *n*_3_. The definition of *Y* and the optimization formula (2) was set to be the same as those given in the white oak trees NGS whole genome shotgun sequencing data analysis.

**Fig 5:**
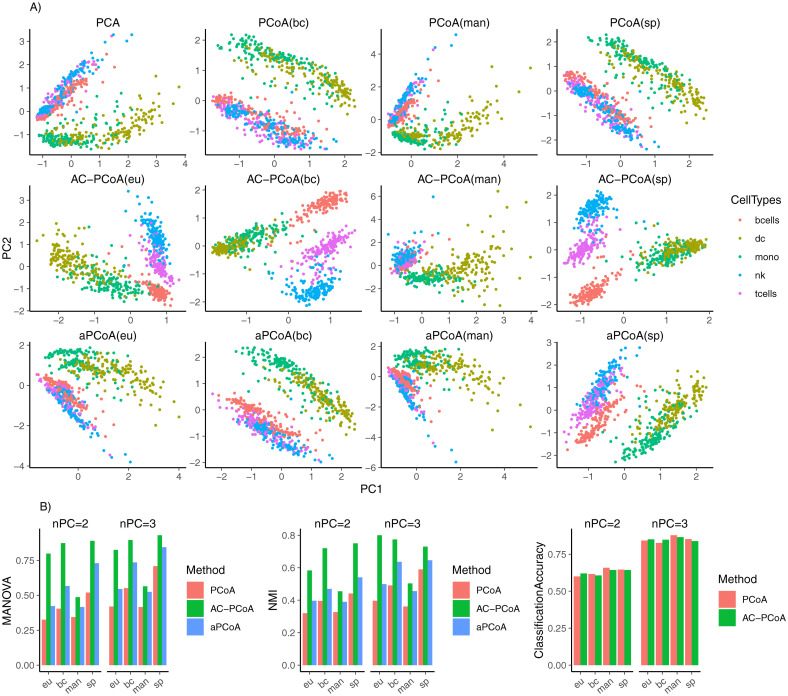
Results of scRNA-Seq data. A: Two-dimensional representations of samples colored by cell types after conducting PCoA, AC-PCoA and aPCoA using four distance measures. B: MANOVA *F*-statistic, NMI of *k*-means clustering, and classification accuracy. Cell types are set to be the true labels. MANOVA, *k*-means clustering, and classification were conducted on two and three principal coordinates from PCoA, AC-PCoA, and aPCoA.

The results are shown in Fig 5. AC-PCoA, including AC-PCA, can better separate different cell types than PCoA and aPCoA in visualization. Bray-Curtis distance and Spearman distance give better results than Euclidean distance in MANOVA *F*-statistic (nPC = 2 and nPC = 3) and NMI (nPC = 2).

Moreover, since tSNE is often employed to perform visualization in single-cell RNA-Seq data analysis, we conducted AC-PCoA to reduce the dimension to 50, and then visualized samples in two-dimensional space using tSNE. We compared the results of tSNE after conducting AC-PCoA to the result of tSNE after conducting PCA and PCoA. The results are plotted in S6 Fig. It shows that AC-PCoA, including AC-PCA, helps to cluster together each cell type.

#### Human brain exon array data

Lastly, we implemented AC-PCoA on a subset of human brain exon array data [39] reported by Lin *et al*. [15]. This dataset includes the transcriptomes of 16 brain regions across developmental epochs. Samples from 10 brain regions in the neocortex were used in the analysis. Lin *et al*. reorganized the data and defined nine time windows by grouping samples from every six donors. By conducting PCA on each donor, they found that the gross morphological structure of the hemisphere was largely recapitulated. This pattern disappeared when PCA was applied to multiple donors in one window simultaneously. When applying AC-PCA, the anatomical structure of neocortex could be recovered since the confounding effects from individual donor were adjusted.

We considered four distance measures: Euclidean distance (eu), Spearman distance (sp), Kendall’s tau (tauD), and Manhattan distance (man). We also performed PCA and AC-PCA on these data to verify the equivalence between PCA and PCoA(eu), and between AC-PCA and AC-PCoA(eu). For one window, let *X*_*i*_ represent the *n* × *p* matrix for the gene expression levels of donor *i*, where *n* is the number of brain regions and *p* is the number of genes. By stacking the rows of *X*_1_, ⋯, *X*_*m*_, where *m* is the number of donors, we obtained the *N* × *p* data matrix *X*, wherein *N* = *n* × *m*. Confounder matrix *Y* was defined to have the same structure as that in the Microbiome Quality Control Project data analysis.

**Fig 6:**
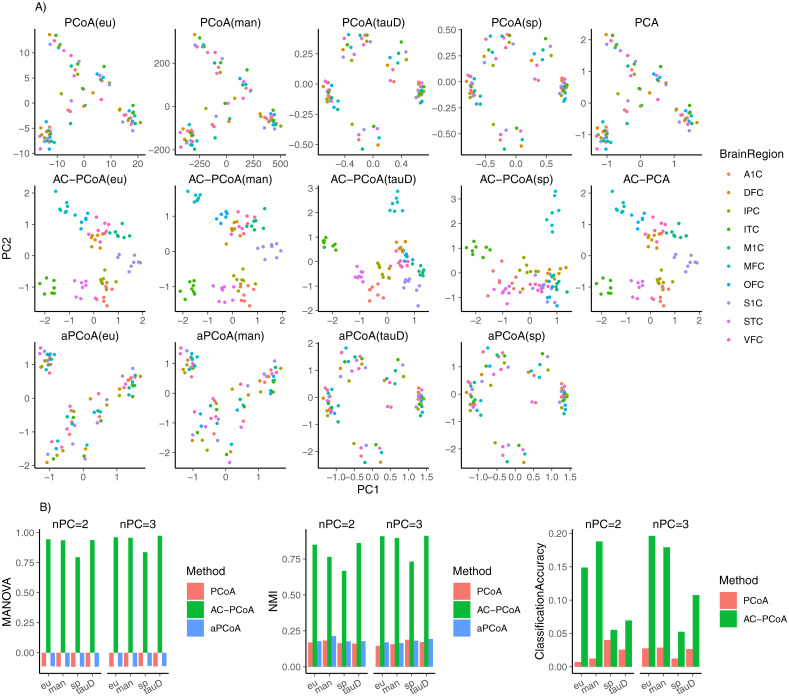
Results of human brain exon array data (window 5). A: Two-dimensional plot colored by brain regions after conducting PCA, AC-PCA, PCoA, AC-PCoA and aPCoA, using four distance measures. B: MANOVA *F*-statistic, NMI of *k*-means clustering, and classification accuracy. Brain regions are set to be the true labels. MANOVA, *k*-means clustering, and classification were conducted on two and three principal coordinates from PCoA, AC-PCoA, and aPCoA.

The results of window 5 are given in Fig 6 as a demonstrating example. Fig 6A shows that the two-dimensional plots of PCA and PCoA(eu) are the same, and the two-dimensional plots of AC-PCA and AC-PCoA(eu) are also the same, thus confirming the equivalence of two-dimensional representations given by AC-PCA and AC-PCoA(eu) in this dataset. In addition to Euclidean distance, Spearman distance, Kendall’s tau distance and Manhattan distance could remove confounding effect and recover the anatomical structure as well. Moreover, aPCoA could not remove the confounding factors in this dataset.

## Discussion

Confounding factors have a significant effect on scientific findings in data-driven research, especially in today’s large-scale data analysis. In this work, we have developed a method called AC-PCoA to simultaneously perform confounding factors adjustment and dimension reduction based on distance measures. AC-PCoA is effective, even when non-Euclidean distance measures are applied to describe pairwise relationships, which is a common case in biological data analysis. AC-PCoA is able to borrow strength from pairwise distances and make use of the underlying topological structures of the samples. Thus, it shows promising results in various kinds of data analysis, especially for data using non-Euclidean distance measures. Practically and significantly, we have showed the good performance of AC-PCoA on the next generation sequencing data, the microbiome taxonomic data, the RNA-Seq data, and the exon array data.

As an exploratory tool, AC-PCoA can be applied in combination with other data analysis methods, such as classification. As shown in the experiments, it can help improve classification accuracy by adjusting for confounding factors. Furthermore, AC-PCoA can be used as a preprocessing step before applying other machine learning methods, such as regression and clustering. Since more and more biological data are used for diagnostic, predictive and classification applications nowadays, it is of paramount importance that AC-PCoA as well as its idea can be further generalized to such scenarios, and even causality analytics [40, 41].

Like most confounding factor adjustment methods, confounding factors are user-defined. In our method, the choices of *Y* and *K* play a crucial role in the whole process. To give proper definitions of *Y* and *K* is not always straightforward. Sometimes researchers have no information at all about the confounding factors. Thus, in our future studies, we will focus much on performing confounding factor adjustment using distance measures with unknown confounding factors.

The R-package with application examples is available at https://github.com/YuWang28/acPCoA.

## Supporting information

S1 Appendix AC-PCoA classification results when nPC is large. (PDF)

S2 Appendix Definitions of distances. (PDF)

S3 Appendix Preprocessing steps for MBQC data. (PDF)

S1 Fig Classification results of PCoA and AC-PCoA when nPC is large, compared with benchmark. (PDF)

S2 Fig MANOVA *F*-statistic of MBQC data (all subsets). (PDF)

S3 Fig *k*-means clustering NMI of MBQC data (all subsets). (PDF)

S4 Fig Classification accuracy of MBQC data (all subsets). (PDF)

S5 Fig SVA results of MBQC data (all subsets). (PDF)

S6 Fig tSNE visualization of scRNA-Seq data after PCoA and AC-PCoA. (PDF)
