# Supplementary material for: AC-PCoA: Adjustment for confounding factors using principal coordinate analysis
Source: PLoS Comput Biol. 2022 Jul 13;18(7):e1010184. doi: 10.1371/journal.pcbi.1010184 (PMC9278763; doi:10.1371/journal.pcbi.1010184)
Supplement: S1 Appendix — (PDF) [file pcbi.1010184.s001.pdf]

AC-PCoA classification results when nPC is large

To use AC-PCoA to enhance classification, we suggest setting the number of principal coordinates large enough to capture most of the information in the data. The results of classification using higher-dimensional representations from AC-PCoA and PCA are shown in S1 Fig. We set the number of PC to be the minimum number needed to explain 90% of the data variance, according to PCA. For comparison, we conducted classification benchmarking by treating the original data and confounder variables as predictors.

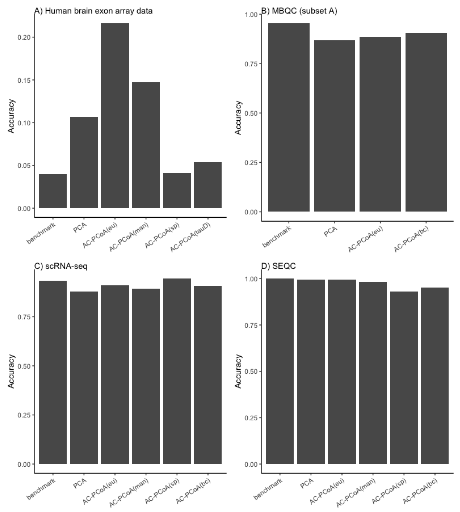

S1 Fig: Classification results of PCoA and AC-PCoA when nPC is large, compared with benchmark. The number of principal coordinates is set to be: A) human brain exon array data: nPC=30. B) MBQC data (subset'A'): nPC=78. C) single cell RNA-Seq data: nPC=542. d) SEQC data: nPC=2.

S1 Fig shows the results of four datasets. AC-PCoA outperforms the benchmark in human brain exon array data and single-cell RNA-Seq data. The performances of AC-PCoA and benchmark are almost the same in MBQC and SEQC data. We did not include tree data because benchmark classification is not applicable to sequence reads.
