# Supplementary material for: AC-PCoA: Adjustment for confounding factors using principal coordinate analysis
Source: PLoS Comput Biol. 2022 Jul 13;18(7):e1010184. doi: 10.1371/journal.pcbi.1010184 (PMC9278763; doi:10.1371/journal.pcbi.1010184)
Supplement: S2 Appendix — (PDF) [file pcbi.1010184.s002.pdf]

Definitions of distances

1 Distances for numerical vectors

Euclidean distance(eu) The Euclidean distance between two points is the length of the line segment connecting them. If $\mathbf{x} = (x_1, x_2, ..., x_n)$ and $\mathbf{y} = (y_1, y_2, ..., y_n)$ are two points in an n-dimensional Euclidean space, then the Euclidean distance between $\mathbf{x}$ and $\mathbf{y}$ is given by

$$d(\mathbf{x}, \mathbf{y}) = \sqrt{\sum_{i=1}^{n} (x_i - y_i)^2}. \quad (1)$$

Manhattan distance(man) Manhattan distance is particular case of the more general Minkowski family of metric distances. For $\mathbf{x} = (x_1, x_2, ..., x_n)$ and $\mathbf{y} = (y_1, y_2, ..., y_n)$, it is defined as

$$d(\mathbf{x}, \mathbf{y}) = \|\mathbf{x} - \mathbf{y}\|_1 = \sum_{i=1}^{n} |x_i - y_i|,$$

Spearman correlation distance(sp) Spearman correlation identifies rank relationships of variables. If both $\mathbf{x}$ and $\mathbf{y}$ are sequences of real numbers in the form $\mathbf{x} = (x_1, x_2, ..., x_n)$ and $\mathbf{y} = (y_1, y_2, ..., y_n)$, Pearson correlation coefficient is defined as

$$r_{xy} = \frac{\sum_{i=1}^{n} (x_i - \bar{x})(y_i - \bar{y})}{\sqrt{\sum_{i=1}^{n} (x_i - \bar{x})^2} \sqrt{\sum_{i=1}^{n} (x_i - \bar{x})^2}}. \quad (2)$$

If the values of each sequence are replaced by their respective ranks, the Spearman correlation coefficient is also given by the above equation. Spearman correlation distance is one minus Spearman correlation coefficient. Given that the actual values of the sequences are replaced by their ranks, Spearman tends to be less sensitive to outliers than Pearson correlation.

Kendall's tau distance(tauD) Kendall's tau(τ) distance is a statistic used to measure the ordinal association between two measured quantities. Let $(x_1, y_1), ..., (x_n, y_n)$ be a set of observations of the joint random variables X and Y, such that all the values of x_i and y_i are unique (ties are neglected for simplicity). Any pair of observations (x_i, y_i) and (x_j, y_j), where $i < j$, are said

to be concordant if the sort order of (x_i, x_j) and (y_i, y_j) agrees: that is, if either both $x_i > x_j$ and $y_i > y_j$ holds or both $x_i < x_j$ and $y_i < y_j$; otherwise they are said to be discordant. The Kendall's tau coefficient is defined as:

$$\tau = \frac{(\text{number of concordant pairs}) - (\text{number of discordant pairs})}{\binom{n}{2}}. \quad (3)$$

Where $\binom{n}{2} = \frac{n(n-1)}{2}$ is the binomial coefficient for the number of ways to choose two items from n items. As the two correlation distance mentioned above, Kendall's tau distance is defined as one minus Kendall's tau rank correlation coefficient.

Bray-Curtis Distance In ecology and biology, the Bray–Curtis dissimilarity, named after J. Roger Bray and John T. Curtis [1], is a statistic used to quantify the compositional dissimilarity between two different sites, based on counts at each site. Here, we computed BC distance by the function "vegdist" in R package "vegan". Let $\mathbf{x} = (x_1, x_2, ..., x_n)$ and $\mathbf{y} = (y_1, y_2, ..., y_n)$ denote the quantity of species in site $\mathbf{x}$ and $\mathbf{y}$, Bray-Curtis Distance between the two sites is given by

$$d(\mathbf{x}, \mathbf{y}) = \frac{\sum_i |x_i - y_i|}{\sum_i (x_i + y_i)} \quad (4)$$

2 Distances for next generation sequences

The following four distance measurements d_2(d2) [6], CVTree [2], d_2^*(d2star) and d_2^s(d2shepp) [3, 4, 5, 7] are designed for NGS data.Given two NGS data sets i and j from different samples and a given word length k, we first count the number of occurrences of all k-mers in all reads of sample i and sample j, respectively. The full set of k-mers of length k is defined as $\mathcal{A}^k$ where $\mathcal{A} = (A, T, C, G)$ for nucleotide sequences. For a given k-mer w, its number of occurrences in data set i is defined as $N_w^{(i)}$ and the frequency or the relative abundance of this k-mer is defined as $f_w^{(i)} = \frac{N_w^{(i)}}{\sum_w N_w^{(i)}}$. We model the background DNA sequence of a sample using m-th order Markov chain where the order m is estimated using the method developed for NGS short read data [4]. The expected number of occurrences of word w, $\mathbb{E}N_w^{(i)}$, can be calculated from the stationary probability of the first m-mer $w[1 : m]$ and the transition probabilities from the n-th m-mer $w[n : n + m - 1]$ to the (n+m)-th nucleotide $w[n + m]$:

$$\mathbb{E}N_w^{(i)} \approx L^{(i)} \mu(w[1 : m]) \prod_{n=1}^{k-m} \pi(w[n : n + m - 1], w[n + m]) \quad (5)$$

where $L^{(i)}$equals to the sum of the lengths of all reads in the i-th data set minus $(m - 1)\mathcal{R}$ where $(m - 1)\mathcal{R}$ is the total number of reads, μ is the stationary probability distribution, and π is the transition probability distribution that can be estimated from the data. The difference between the number of occurrences of

k-mer w and its expected number of occurrences is defined as $\tilde{N}_w^{(i)} = N_w^{(i)} - \mathbb{E}N_w^{(i)}$ that we refer to as the background adjusted k-mer counts.

d_2**(d2)**

$$d_2 = \frac{1}{2}\left(1 - \frac{\sum_{w\in\mathcal{A}^k} f_w^{(i)} f_w^{(j)}}{\sqrt{\sum_{w\in\mathcal{A}^k}(f_w^{(i)})^2}\sqrt{\sum_{w\in\mathcal{A}^k}(f_w^{(j)})^2}}\right) \quad (6)$$

CVTree

$$CVTree = \frac{1}{2}\left(1 - \frac{\sum_{w\in\mathcal{A}^k} \hat{f}_w^{(i)} \hat{f}_w^{(j)}}{\sqrt{\sum_{w\in\mathcal{A}^k}(\hat{f}_w^{(i)})^2]}\sqrt{\sum_{w\in\mathcal{A}^k}(\hat{f}_w^{(j)})^2}}\right) \quad (7)$$

where $\hat{f}_w^{(i)} = \frac{\hat{N}_w^{(i)}}{\mathbb{E}N_w^{(i)}}$. CVTree calculates $\mathbb{E}\hat{N}_w^{(i)}$ by assuming a (k-2)-th order Markov chain for genomic sequences

d_2∗**(d2star)**

$$CVTree = \frac{1}{2}\left(1 - \frac{\sum_{w\in\mathcal{A}^k} \bar{f}_w^{(i)} \bar{f}_w^{(j)}}{\sqrt{\sum_{w\in\mathcal{A}^k}(\bar{f}_w^{(i)})^2}\sqrt{\sum_{w\in\mathcal{A}^k}(\bar{f}_w^{(j)})^2}}\right) \quad (8)$$

where $\bar{f}_w^{(i)} = \frac{\hat{N}_w^{(i)}}{\sqrt{\mathbb{E}N_w^{(i)}}}$.

d_2^s**(d2shepp)**

$$CVTree = \frac{1}{2}\left(1 - \frac{\sum_{w\in\mathcal{A}^k} \tilde{f}_w^{(i)} \tilde{f}_w^{(j)}}{\sqrt{\sum_{w\in\mathcal{A}^k}(\tilde{f}_w^{(i)})^2}\sqrt{\sum_{w\in\mathcal{A}^k}(\tilde{f}_w^{(j)})^2}}\right) \quad (9)$$

where $\tilde{f}_w^{(i)} = \frac{\hat{N}_w^{(i)}}{((\tilde{N}_w^{(i)})^2 + (\tilde{N}_w^{(j)})^2)^{\frac{1}{4}}}$ and $\tilde{f}_w^{(j)} = \frac{`A', `B', `C', `D', `E', `F'\hat{N}_w^{(j)}}{((\tilde{N}_w^{(i)})^2 + (\tilde{N}_w^{(j)})^2)^{\frac{1}{4}}}$

References

[1] John R Bray and John T Curtis. An ordination of the upland forest communities of southern wisconsin. *Ecological monographs*, 27(4):325–349, 1957.

[2] Ji Qi, Hong Luo, and Bailin Hao. Cvtree: a phylogenetic tree reconstruction tool based on whole genomes. *Nucleic acids research*, 32(suppl_2):W45–W47, 2004.

[3] Gesine Reinert, David Chew, Fengzhu Sun, and Michael S Waterman. Alignment-free sequence comparison (i): statistics and power. *Journal of Computational Biology*, 16(12):1615–1634, 2009.

[4] Jie Ren, Kai Song, Minghua Deng, Gesine Reinert, Charles H Cannon, and Fengzhu Sun. Inference of markovian properties of molecular sequences from ngs data and applications to comparative genomics. *Bioinformatics*, 32(7):993–1000, 2016.

[5] Kai Song, Jie Ren, Zhiyuan Zhai, Xuemei Liu, Minghua Deng, and Fengzhu Sun. Alignment-free sequence comparison based on next-generation sequencing reads. *Journal of computational biology*, 20(2):64–79, 2013.

[6] David C Torney, Christian Burks, Daniel Davison, and Karl M Sirotkin. Computation of d 2: A measure of sequence dissimilarity. In *Computers and DNA*, pages 109–125. Routledge, 2018.

[7] Lin Wan, Gesine Reinert, Fengzhu Sun, and Michael S Waterman. Alignment-free sequence comparison (ii): theoretical power of comparison statistics. *Journal of Computational Biology*, 17(11):1467–1490, 2010.
