# Supplementary material for: AC-PCoA: Adjustment for confounding factors using principal coordinate analysis
Source: PLoS Comput Biol. 2022 Jul 13;18(7):e1010184. doi: 10.1371/journal.pcbi.1010184 (PMC9278763; doi:10.1371/journal.pcbi.1010184)
Supplement: S3 Appendix — (PDF) [file pcbi.1010184.s003.pdf]

Preprocessing steps of MBQC data

Subset 'A', 'B', 'C', 'D', 'E', 'F' We generated subset 'A',$\cdots$, 'F' by choosing samples processed by their own biology lab and different bioinformatics labs. Let us take bioinformatics lab 'A' as an example. We randomly chose 4-5 samples from each specimen. Hence we have 106 samples. Each of these samples was then processed by 8 bioinformatics labs, resulting in 848 samples in total.

Mathematically, let X_i^A represent the $n \times p$ matrix for OTU levels of samples processed by biology lab A and bioinformatics lab i where n is the number of samples and p is the number of OTUs. For all bioinformatics lab i, note that X_i^A contains the same number of samples n. Thus, the kth sample in X_i^A and the kth sample in X_j^A have the same origin from biology lab A. By stacking the rows of $X_1^A, \cdots, X_8^A$, we formed a $(8n) \times p$ matrix, X^A, representing the subset of data from biology lab 'A'. Similarly, we constructed the data matrix of subset 'B', 'C', 'D', 'E', and 'F', denoted as $\{X^A, X^B, X^C, X^D, X^E, X^F\}$. Our notation of subsets corresponds to the notation of labs in the original data in the following way: 'A' to HL-B, 'B' to HL-C, 'C' to HL-E, 'D' to HL-F, 'E' to HL-H, and 'F' to HL-J. We used X as a generic notation of the data from the above subsets and defined X_i and X_j to be the submatrices of X representing samples from bioinformatics lab i and j.

The confounders are defined to be the difference between different bioinformatics labs. By choosing linear kernel and defining Y to have only two non-zero entries in each column ,1 and -1, corresponding to the row of a pair of samples from the same specimen but different labs, the objective function Equation(2) in the main text is defined as follows:

$$\max_{||\boldsymbol{v}||_2^2 \leq 1} \boldsymbol{v}^T \hat{X}^T \hat{X} \boldsymbol{v} - \frac{\lambda}{8} \sum_{i=1}^{8-1} \sum_{j=i+1}^{8} \boldsymbol{v}^T (\hat{X}_j - \hat{X}_i)^T (\hat{X}_j - \hat{X}_i) \boldsymbol{v}$$

where $\hat{X}$ is the approximate Euclidean representation of X.

Subset'1', '2', '3', '4', '5', '6', '7', '8' We generated subset '1',$\cdots$, '8' by choosing samples processed by their own biology lab and different bioinformatics labs. Let us take subset '1' as an example. We first considered all samples processed by bioinformatics lab 1. Among these samples, we selected 0-60 samples sequenced by each biology lab i, $i = A \cdots F$, which may have originally been from different specimens. Note that this number does not need to be exactly the same for each biology labs, owing to the limitation of original data.

Mathematically, let X_{il}^1 represent the $n_{il} \times p$ matrix for OTU levels of samples of specimen l from biology lab i where n_{il} is the number of samples and p is the number of OTUs. By stacking the rows of $X_{i1}^1, \cdots, X_{i22}^1$, we got an $n_i \times p$ matrix X_i^1 representing OTU levels of samples from biology lab i where $n_i = \sum_{k=1}^{22} n_{ik}$. By further stacking the rows of $X_1^1, \cdots, X_6^1$, we had an $N_1 \times p$ matrix X^1, representing the whole subset of data '1', where $N_1 = n_1 + n_2 + n_3 + n_4 + n_5 + n_6$. Similarly, we defined data matrix of subset '1',$\cdots$, '8', denoted$\{X^1, \cdots, X^8\}$. Our notation of subsets corresponds to the notation of labs in the original data in the following way: '1' to BL-1, '2' to BL-2, '3' to BL-3, '4' to BL-4, '5' to BL-6, '6' to BL-8, '7' to BL-9A, and '8' to BL-9B. We also use X as a generic notation of data and define X_{il} to be the sub matrix of X, representing samples of specimen l from biology lab i.

The confounders in this setting are defined to be the differences among biology labs. We chose linear kernel and defined each column of Y to have only two groups of non-zero entries, $\frac{1}{n_{ik}}$ and $-\frac{1}{n_{jk}}$, corresponding to the rows of samples of specimen k from biology lab i and j. The objective function of Equation(2) in the main text is defined as follows:

$$\max_{||\boldsymbol{v}||_2^2 \leq 1} \boldsymbol{v}^T \hat{X}^T \hat{X} \boldsymbol{v} - \frac{\lambda}{6} \sum_{i=1}^{6-1} \sum_{j=i+1}^{6} \sum_{l=1}^{22} \boldsymbol{v}^T (\bar{X}_{jl} - \bar{X}_{il})^T (\bar{X}_{jl} - \bar{X}_{il}) \boldsymbol{v}$$

where $\hat{X}$ is the approximate Euclidean representation of X, and $\bar{X}_{ik}$ is a $1 \times p$ matrix containing the row mean of matrix $\hat{X}_{ik}$.
