# Supplementary material for: AC-PCoA: Adjustment for confounding factors using principal coordinate analysis
Source: PLoS Comput Biol. 2022 Jul 13;18(7):e1010184. doi: 10.1371/journal.pcbi.1010184 (PMC9278763; doi:10.1371/journal.pcbi.1010184)
Supplement: S1 Fig — (PDF) [file pcbi.1010184.s004.pdf]

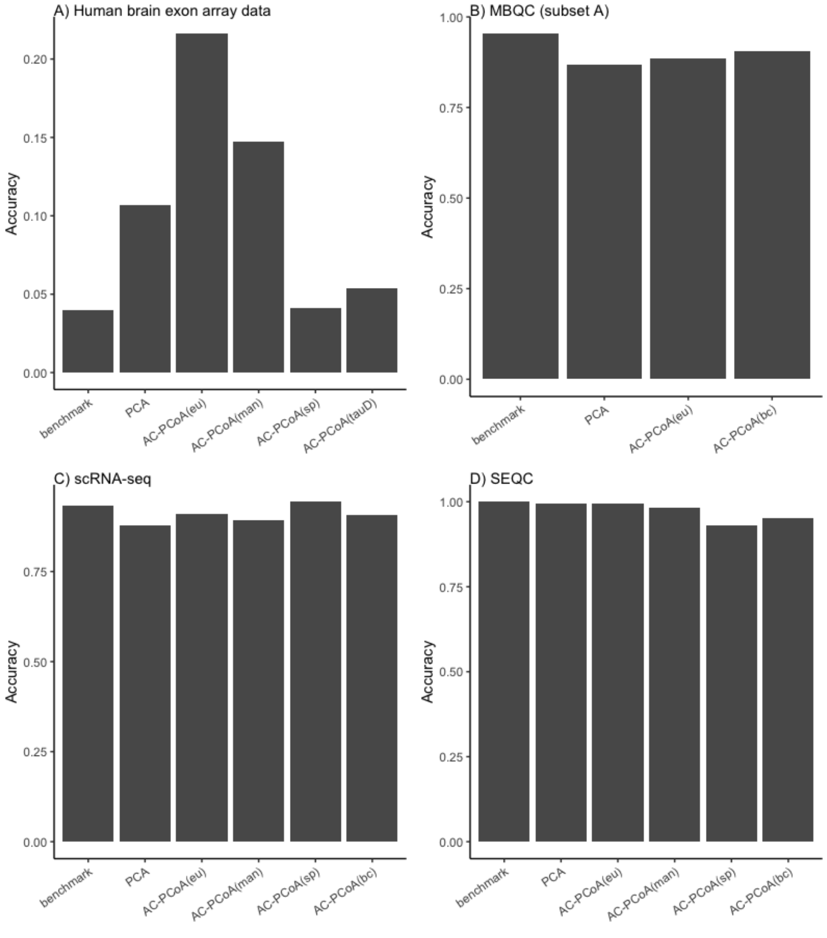

S1 Fig: Classification results of PCoA and AC-PCoA when nPC is large, compared with benchmark. The number of principal coordinates is set to be: A: human brain exon array data: nPC=30. B: MBQC data (subset'A'): nPC=78. C: single cell RNA-Seq data: nPC=542. D: SEQC data: nPC=2.
