# Supplementary material for: AC-PCoA: Adjustment for confounding factors using principal coordinate analysis
Source: PLoS Comput Biol. 2022 Jul 13;18(7):e1010184. doi: 10.1371/journal.pcbi.1010184 (PMC9278763; doi:10.1371/journal.pcbi.1010184)
Supplement: S2 Fig — (PDF) [file pcbi.1010184.s005.pdf]

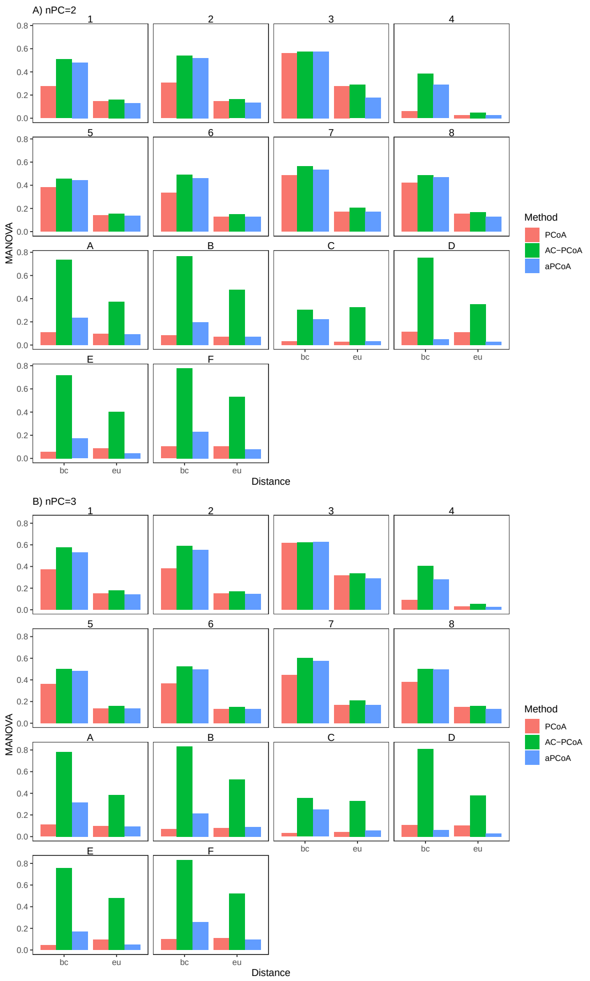

S2 Fig: MANOVA *F*-statistic of MBQC data (all subsets). MANOVA *F*-statistic on A: two- and B: three-dimensional plot from PCoA, AC-PCoA and aPCoA. Specimens are set to be the true labels. 14 subsets '1', '2', '3', '4', '5', '6', '7', '8', 'A', 'B', 'C', 'D', 'E', 'F' are included.
