# Supplementary material for: AC-PCoA: Adjustment for confounding factors using principal coordinate analysis
Source: PLoS Comput Biol. 2022 Jul 13;18(7):e1010184. doi: 10.1371/journal.pcbi.1010184 (PMC9278763; doi:10.1371/journal.pcbi.1010184)
Supplement: S3 Fig — (PDF) [file pcbi.1010184.s006.pdf]

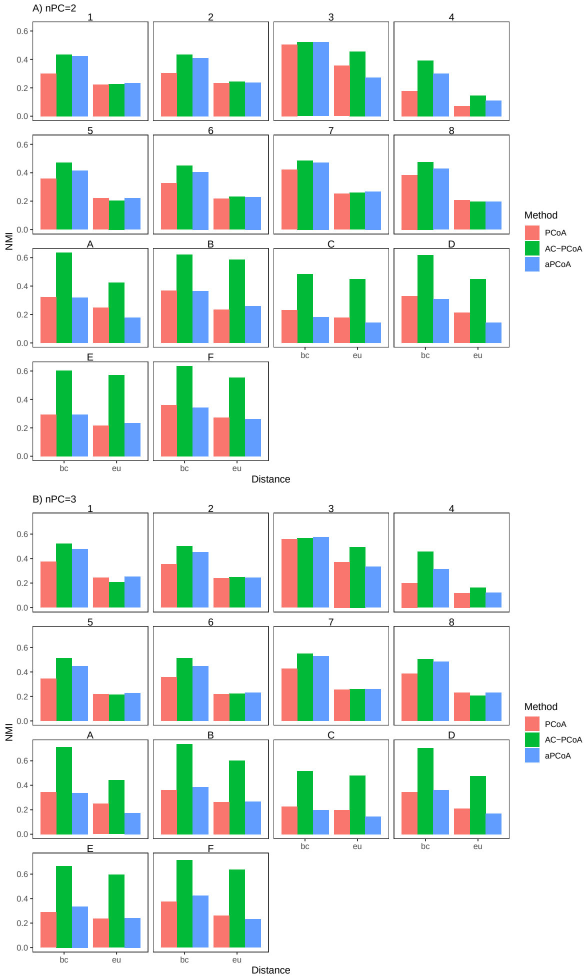

S3 Fig: k-means clustering NMI of MBQC data (all subsets). NMI of k-means clustering of A: two and B: three principal coordinates from PCoA, AC-PCoA and aPCoA with for 14 subsets ‘1’, ‘2’, ‘3’, ‘4’, ‘5’, ‘6’, ‘7’, ‘8’, ‘A’, ‘B’, ‘C’, ‘D’, ‘E’, ‘F’. Specimens were set to be the true labels. The number of clusters k in k-means was set as the number of specimens.
