# Supplementary material for: AC-PCoA: Adjustment for confounding factors using principal coordinate analysis
Source: PLoS Comput Biol. 2022 Jul 13;18(7):e1010184. doi: 10.1371/journal.pcbi.1010184 (PMC9278763; doi:10.1371/journal.pcbi.1010184)
Supplement: S4 Fig — (PDF) [file pcbi.1010184.s007.pdf]

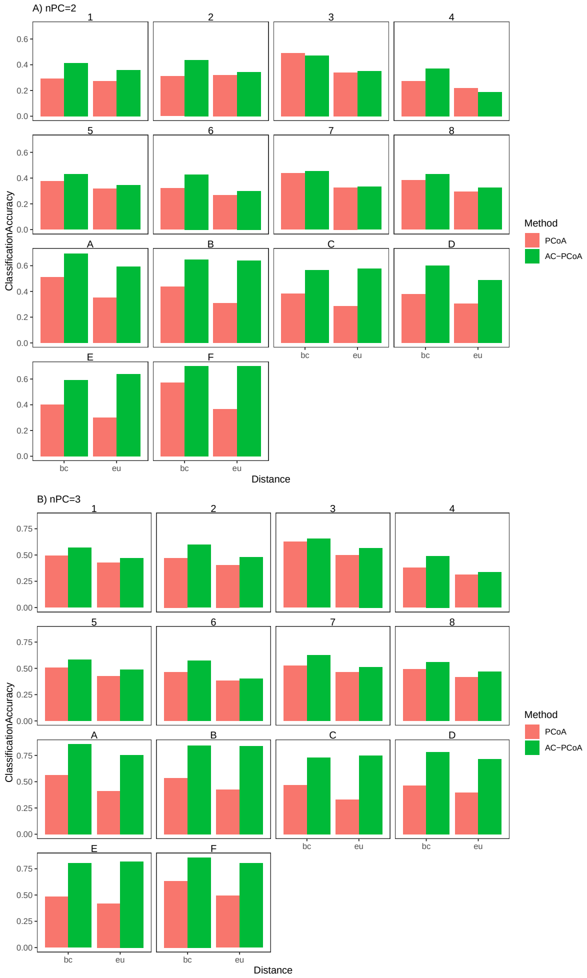

S4 Fig: Classification accuracy of MBQC data (all subsets). Classification accuracy on A: two and B: three principal coordinates from PCoA and AC-PCoA for 14 subsets '1', '2', '3', '4', '5', '6', '7', '8', 'A', 'B', 'C', 'D', 'E', 'F'. Specimens were set to be the true labels. Five fold cross validation were performed using Random Forest as classifier.
