# Supplementary material for: AC-PCoA: Adjustment for confounding factors using principal coordinate analysis
Source: PLoS Comput Biol. 2022 Jul 13;18(7):e1010184. doi: 10.1371/journal.pcbi.1010184 (PMC9278763; doi:10.1371/journal.pcbi.1010184)
Supplement: S5 Fig — (PDF) [file pcbi.1010184.s008.pdf]

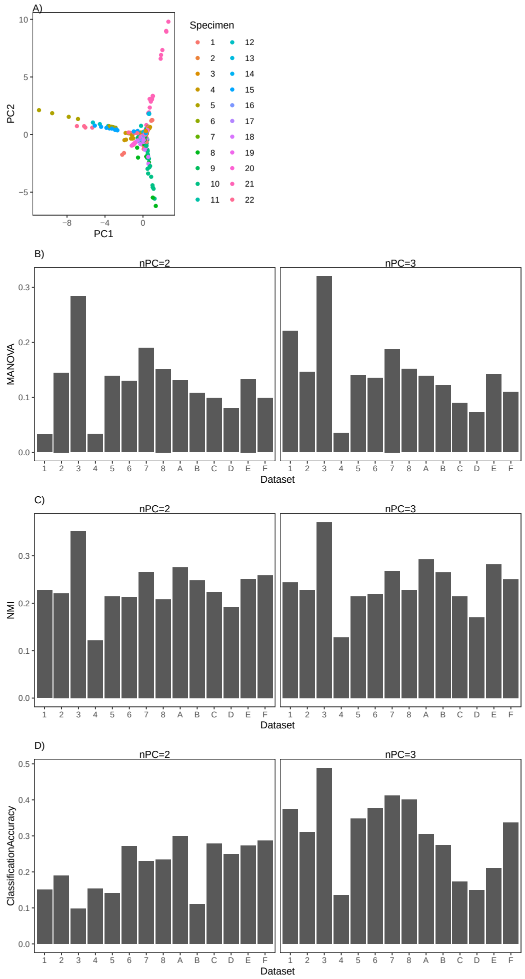

S5 Fig: SVA results of MBQC data (all subsets). Perform SVA to remove confounding factors and then apply PCA to do visualization. Results of 14 subsets ‘1’, ‘2’, ‘3’, ‘4’, ‘5’, ‘6’, ‘7’, ‘8’, ‘A’, ‘B’, ‘C’, ‘D’, ‘E’, ‘F’ are included. Specimens are set to be the true labels. A: Two-dimensional plots of SVA and PCA on representative dataset ‘A’. B: MANOVA F-statistic on two and three principal components from PCA based on SVA results. C: NMI of k-means clustering. D: Classification accuracy.
