# Supplementary material for: AC-PCoA: Adjustment for confounding factors using principal coordinate analysis
Source: PLoS Comput Biol. 2022 Jul 13;18(7):e1010184. doi: 10.1371/journal.pcbi.1010184 (PMC9278763; doi:10.1371/journal.pcbi.1010184)
Supplement: S6 Fig — (PDF) [file pcbi.1010184.s009.pdf]

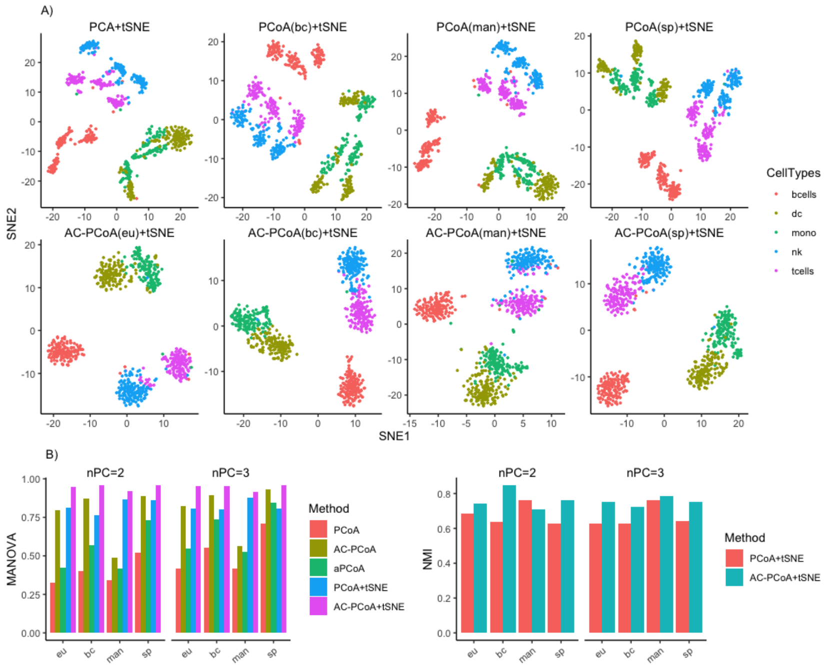

S6 Fig: tSNE visulization of scRNA-Seq data after PCoA and AC-PCoA. A: tSNE visualization on the first 50 principal coordinates from PCoA and AC-PCoA. Cells are colored by cell types. B: MANOVA F-statistic and NMI of k-means clustering on two- and three-dimensional representations of cells given by PCoA and AC-PCoA. Cell types are set to be the true labels.
